# Supplementary material for: Complex‐centric proteome profiling by SEC‐SWATH‐MS
Source: Mol Syst Biol. 2019 Jan 14;15(1):e8438. doi: 10.15252/msb.20188438 (PMC6346213; doi:10.15252/msb.20188438)
Supplement: Supplementary file 8 — Dataset EV7 [file MSB-15-e8438-s008.zip › feature_plots_string/Q15118.pdf]

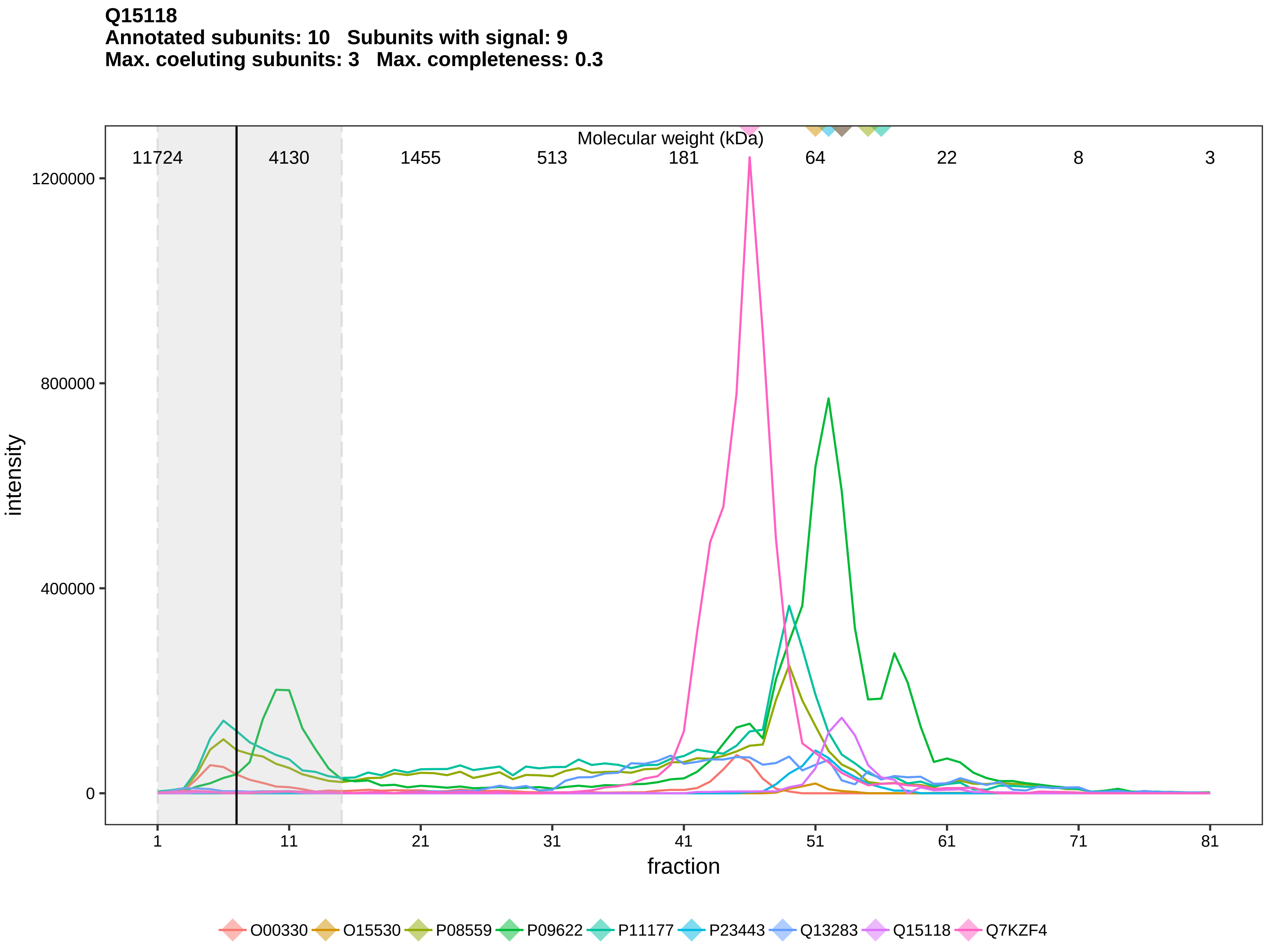
Q15118
Annotated subunits: 10 Subunits with signal: 9
Max. coeluting subunits: 3 Max. completeness: 0.3
Molecular weight (kDa)
11724
4130
1455
513
181
64
22
8
3
1200000
800000
400000
0
intensity
1
11
21
31
41
51
61
71
81
fraction
O00330
O15530
P08559
P09622
P11177
P23443
Q13283
Q15118
Q7KZF4
